# Dual thio-digalactoside-binding modes of human galectins as the structural basis for the design of potent and selective inhibitors

**DOI:** 10.1038/srep29457

**Published:** 2016-07-15

**Authors:** Tung-Ju Hsieh, Hsien-Ya Lin, Zhijay Tu, Ting-Chien Lin, Shang-Chuen Wu, Yu-Yao Tseng, Fu-Tong Liu, Shang-Te Danny Hsu, Chun-Hung Lin

**Affiliations:** 1 Institute of Biological Chemistry, Academia Sinica, Taipei, 11529, Taiwan; 2 Department of Chemistry, National Taiwan University, Taipei, 10617, Taiwan; 3 Institute of Biochemical Sciences, National Taiwan University, Taipei, 10617, Taiwan; 4 Institute of Biomedical Sciences, Academia Sinica, Taipei, 11529, Taiwan; 5 The Genomics Research Center, Academia Sinica, Taipei, 11529, Taiwan

## Abstract

Human galectins are promising targets for cancer immunotherapeutic and fibrotic disease-related drugs. We report herein the binding interactions of three thio-digalactosides (TDGs) including TDG itself, TD139 (3,3’-deoxy-3,3’-bis-(4-[*m*-fluorophenyl]-1H-1,2,3-triazol-1-yl)-thio-digalactoside, recently approved for the treatment of idiopathic pulmonary fibrosis), and TAZTDG (3-deoxy-3-(4-[*m*-fluorophenyl]-1H-1,2,3-triazol-1-yl)-thio-digalactoside) with human galectins-1, -3 and -7 as assessed by X-ray crystallography, isothermal titration calorimetry and NMR spectroscopy. Five binding subsites (A–E) make up the carbohydrate-recognition domains of these galectins. We identified novel interactions between an arginine within subsite E of the galectins and an arene group in the ligands. In addition to the interactions contributed by the galactosyl sugar residues bound at subsites C and D, the fluorophenyl group of TAZTDG preferentially bound to subsite B in galectin-3, whereas the same group favored binding at subsite E in galectins-1 and -7. The characterised dual binding modes demonstrate how binding potency, reported as decreased *K*_*d*_ values of the TDG inhibitors from μM to nM, is improved and also offer insights to development of selective inhibitors for individual galectins.

Galectins are small, soluble lectins characterised by conserved β-galactoside-binding site (subsites C and D) within their carbohydrate-recognition domains (CRD, ~130 amino acid residues), and they can form multivalent lattices with membrane-associated glycosylated ligands^1^,^2^,^3^. Although all natural, endogenous ligands of galectins and their corresponding biological roles remain to be elucidated, their glycan-binding activities have been shown to control the concentrations, localisation, and availabilities of glycoproteins (including glycosylated receptors) on the cell surface, thereby allowing galectins to modulate the transmembrane signalling events of diverse physiological and pathological processes, e.g., cell adhesion, proliferation, differentiation as well as inflammation, angiogenesis, cancer progression and metastasis^4^,^5^,^6^. Overexpression of specific galectins has been associated with neoplastic transformation, poor cancer-related outcomes and progressive fibrosis during organ failure, supporting the idea that newly developed anti-galectin agents will be useful as cancer immunotherapeutics and for fibrotic disease therapies^7^,^8^,^9^,^10^. All the observed CRDs of galectin family adopt a typical β-sandwich fold composed of two antiparallel β-sheets of six strands (S1–S6, S-sheet) and five strands (F1–F5, F-sheet). Concave surface of S-sheet contains conserved amino residues and forms a primary binding groove to which specific glycans up to a length of tetrasaccharide are bound^11^,^12^. To orientate each sugar residue, the CRD groove was described in terms of the subsites A–E (Figure S1)^12^,^13^,^14^. In this model, the best structurally characterised subsites C and D are responsible for recognition of the β-galactoside-containing disaccharides, whereas the other subsites (A, B and E) remain poorly understood regarding how they contribute to ligand binding interactions. A variety of chemical scaffolds have been exploited for the design of promising anti-galectin agents^8^,^15^,^16^. Notably, derivatives of the thio-digalactoside (TDG) scaffold, which is resistant to hydrolysis, have substantial affinity for several galectins^14^,^17^,^18^,^19^. Specifically, these TDG derivatives bear two identical or different substituents at their C3-/C3′-positions, i.e., they are C2-symmetric or C2-asymmetric compounds, respectively. Among them, 3,3′-deoxy-3,3′-bis-(4-[*m*-fluorophenyl]-1H-1,2,3-triazol-1-yl)-thio-digalactoside (TD139; Fig. 1) is the most potent inhibitor of galectins-1 and -3^9^,^20^, and has been approved by the United States Food and Drug Administration for the treatment of idiopathic pulmonary fibrosis. *In silico* computational studies of TD139/galectin-3, based on the X-ray crystal structures of galectin-3 in complex with TDG^17^,^18^ or 3′-(4-methoxy-2,3,5,6-tetrafluorobenzamido)-N-acetyl-lactosamine (L3)^21^ (Fig. 1), indicate that the thio-digalactoside moiety is situated at subsites C and D of the galectin CRD. According to the computational studies, the two TD139 aromatic substituents likely stack intermolecularly with adjacent arginines (Arg144^hGal3^ and Arg186^hGal3^) at subsites B and E of galectin-3, respectively, providing π-cation interactions^22^,^23^,^24^, and could account for its enhanced binding affinity. However, direct structural information concerning subsite E-ligand interactions is not available because previous studies focused on the optimisation of ligand binding at subsites B, C, and D.

Multiple sequence alignments for human galectins-1 to -12 have shown that the majority contains no more than two total arginines at subsites B and E, except for galectin-10, and C-terminal CRD of galectins-4 and -12 where there are none arginines at subsites B and E (Figure S2). Therefore, subsites B and E might provide the increased binding affinity of TD139 when both subsites contain Arg residues. We therefore prepare TDG, TD139 and TAZTDG (C2-asymmetric, containing one 4-fluorophenyl-triazole at C3; Fig. 1) and study their binding interactions with human galectins-1, -3 and -7 by X-ray crystallography, isothermal titration calorimetry (ITC) and NMR spectroscopy. Galectin-1 has one arginine (Arg73^hGal1^) at subsite E and none at subsite B, whereas galectins-3 and -7 contain an arginine at both subsites. TD139 potently inhibits galectins-1 and -3, but not galectin-7^9^. We show that a great number of interactions between TD139 and galectins-1 and -7 exist in subsite E than in subsite B, and that TAZTDG displays two binding modes toward the galectins, with a preference for subsites C–E in galectins-1 and -7 and subsites B–D in galectin-3. In addition to demonstrating how the affnity can be improved >1000-fold, such information provides valuable insights for the design of potent and selective inhibitors for specific galectins.

## Results and Discussion

### Binding affinity analysis of TDG and derivatives for the three galectins

Because the three inhibitors share the same thio-digalactoside core and differ only according to the number of [3-deoxy-3-(4-[*m*-fluorophenyl]-1H-1,2,3-triazol-1-yl) substitutents, assessment by ITC allowed us to characterise how the substitutents affected the binding affinities of the inhibitors with galectins-1, -3 and -7. The resulting values of *K*_*d*_, ΔG, ΔH, ΔS and n (stochiometry) are summarised in Table 1. In all cases, the n-values indicate an 1:1 ratio of galectin/inhibitor. Specifically, TD139 displays the highest binding affinity for galectin-3 (*K*_*d*_ = 68 nM), an ~1000-fold binding enhancement compared with that for TDG (*K*_*d*_ = 75 μM), which contains no substituents. The enhanced binding affinity corresponds to a change in binding free energy (ΔΔG_TD139-TDG_) of −4.3 kcal/mol, which is contributed by large enthalpy change (ΔΔH_TD139-TDG_ = −8.8 kcal/mol) and includes an unfavorable entropy change (–TΔΔS_TD139-TDG_ = 4.6 kcal/mol). Other ΔΔH values for the inhibitors and galectin-3 are ΔΔH_TAZTDG–TDG_ = −5.3 kcal/mol and ΔΔH_TD139–TAZTDG_ = −3.5 kcal/mol. The changes in the ΔΔH values correlate with incorporation of an addition 4-fluorophenyl-triazole at C3 (or C3′), and we assume these changes to be a consequence of an arginine-arene interaction at subsite B or E^22^,^23^. For galectins-1 and -7, the change in binding enthalpy is substantial only for the addition of one sbstituent (ΔΔH_TAZTDG–TDG_ = −4.5 and −2.4 kcal/mol, respectively), whereas the ΔΔH_TD139–TAZTDG_ (=−0.6 and 0.7 kcal/mol, respectively) is much smaller when the second substituent is added.

Galectin-7 contains two arginines (Arg31^hGal7^ and Arg74^hGal7^) at the same polypeptide sequence positions as in galectin-3 (Arg144^hGal3^ and Arg186^hGal3^) (Figure S2). Therefore, the binding affinities of galectin-7 for the three inhibitors were expected to be similar to those of galectin-3 if π-cation interactions were present. However, TD139 bound to galectin-7 with only a 15-fold increase in affinity compared with TDG binding, in correspondence to a small difference in the binding free energy (ΔΔG_TD139-TDG_) of −1.7 kcal/mol. This relatively small increase contrasts with the ~300-fold binding enhancement measured for galectin-1 binding to TD139 compared with binding to TDG; galectin-1 has only an arginine (Arg73^hGal1^) at subsite E (Figure S2). Structural studies were therefore required to characterise the relationships between the substituents and the galectin-binding sites.

### Crystal structures of galectins-1, -3CRD and galectin-7 in complex with TD139

**Figure 2:**
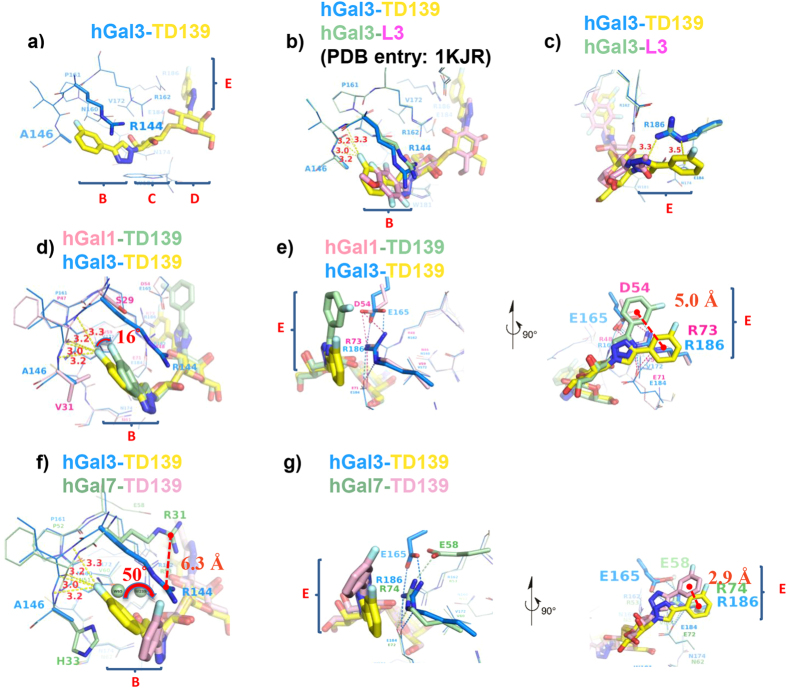
Structural comparison of the carbohydrate recognition domains of galectins in complex with L3 and TD139. (**a**) Overall view of ligand binding site of TD139/galectin-3CRD complex shown in a stick model. Subsites B–E are labelled according to the carbohydrate recognition domain of galectin members. Close-up views of L3/galectin-3CRD (**b**,**c**), TD139/galectin-1 (**d**,**e**), and TD139/galectin-7 (**f**,**g**) superpositioned onto the TD139/galectin-3CRD subsites B and E. (**b**,**d**,**f**) Multiple orthogonal polar interactions between the fluorine atom of 4-fluorophenyl group and the backbone peptide atoms of the galectins are shown as yellow dashed lines. (**c**) The tandem π-arginine interaction between Arg186^hGal3^ and the TD139 substituent is highlighted as yellow dashed lines. (**e**,**g**) Two orthogonal views (Left: side-view, Right: top-view) show the distinct tandem π-ionic interactions in the galectins-1, -3 and -7 complexes. The salt bridge networks of galectins-1, -3 and -7, including the Arg73^hGal1^/144^hGal3^/74^hGal7^ residues in subsite E, are highlighted as pink, blue and green dashed lines, respectively.

The TD139/galectin crystal structures were determined by molecular replacement method using ligand-free galectin structures as the search model (Figure S3 and Table S1). Protein-ligand interface analyses (PDBePISA) performed on all the complex structures indicate that galectin-1 provides the most buried surface area for TD139 binding (383 Å^2^) than galectin-3 (372 Å^2^) and galectin-7 (285 Å^2^). The electron density of TD139 in each complex is clearly visible in its Fo-Fc electron density map (Figure S3d–f). The average B-factor for residues interacting with TD139 (24 Å^2^) is less than the average B-factor for all protein residues (32 Å^2^), which allowed for unambiguous modelling of the geometries of the residues in galectins-1, -3 and -7 that interact with TD139. The TD139/galectin-3 complex had the greatest number of binding interactions (similar to the number for the L3/galectin-3 complex^21^), including salt bridge networks and van der Waals contacts in the principal β-galactoside-recognition subsites C and D (Figure S3h). For the TD139 and L3 complexes, however, different protein-ligand interactions occurred at subsites B and E (Fig. 2a–c). Specifically, although the aromatic amide of L3 and the 4-fluorophenyl moiety of TD139 are both sandwiched between Arg144^hGal3^ and Ala146^hGal3^ in subsite B (Fig. 2b), the TD139 substituent extends deeper into the pocket formed by Arg144^hGal3^ and Ala146^hGal3^ (Fig. 2b). A fluorophilic microenvironment^25^,^26^,^27^ is thereby formed in which the terminal fluorine atom of TD139 can form multiple, orthogonal polar interactions (namely fluorine bondings) with the protein backbone atoms at an average distance <3.5 Å (Fig. 2b). A displacement of ~0.9 Å of the central carbon (C_ζ_) of Arg144^hGal3^ guanidino group seems to accommodate the longer 4-fluorophenyl-triazole substituent of TD139. Moreover, the 4-fluorophenyl and triazole moieties at the other end of TD139 form tandem arginine-π interactions with Arg186^hGal3^ at subsite E (Fig. 2c). The fluorophilic interactions and the arginine-π interaction in subsite E probably account for the 50-fold increase in binding affinity for galectin-3 as compared with that of L3 (*K*_*d*_ = 3.3 μM)^21^,^28^.

In galectin-1, Ser29 and Val31 correspond to Arg144^hGal3^ and Ala146^hGal3^. Even though the 4-fluorophenyl moiety appears to fit in a similar manner into the pocket formed by Ser29^hGal1^ and Val31^hGal1^ (Fig. 2d), the substituent is displaced from Val31^hGal1^ by ~16°, resulting in the formation of only two fluorine-protein interactions in comparison with four observed in the galectin-3 complex. Galectin-7 contains Arg31 and His33 at the positions held by Arg144^hGal3^ and Ala146^hGal3^, but Arg31^hGal7^ is displaced in subsite B and thus does not interact with the 4-fluorophenyl substituent of TD139 (Fig. 2f). Likely hindered by the imidazole of His33^hGal7^, a much bulkier residue than the counterparts Val31^hGal1^ and Ala146^hGal3^, the 4-fluorophenyl moiety turns ~50° away as compared to that in the galectin-3 complex (Fig. 2f), and vacated volume in subsite B of galectin-7 is occupied by two water molecules (Fig. 2f and Figure S2i).

The manner in which the 4-fluorophenyl-triazole moiety of TD139 is situated in subsite E is a consequence of the salt-bridge networks of galectins (Fig. 2e,g)^11^, i.e., Asp54^hGal1^-Arg73^hGal1^-Glu71^hGal1^, Glu165^hGal3^-Arg186^hGal3^-Glu184^hGal3^, and Glu58^hGal7^-Arg74^hGal7^-Glu72^hGal7^. That is to say, the network orients the Arg residue to interact with the aromatic substituent. In the complex structures of galectins-3 and -7, there are similar tandem arginine-π interactions between the 4-fluorophenyl-triazole and Arg186^hGal3^/Arg74^hGal7^ (Fig. 2g). Because the 4-fluorophenyl-trizole moiety of TD139 is found to flip over at substie E of galectin-1, the aromatic substituent becomes close to Asp54^hGal1^ for formation of anion-π interaction (Fig. 2e), which is favored by the fluorinated arene (i.e., an electron-deficient π system)^29^,^30^. Taken together, the comparison among these galectin/TD139 complexes indicates that the common arginine-arene interactions at subsite E are substantial, despite the small differences in their respective interaction geometries (Fig. 2e,g). By contrast, the interactions between TD139 and the B subsite in the galectins differ such that the number of interactions decrease as: galectin-3 > galectin-1 > galectin-7. Especially for galectins-1 and -7, subsite B is less important for binding than is subsite E. Thio-digalactoside moiety of TAZTDG is thus supposed to bind with subsites C and D of galectins-1 and -7, and their E subsites accommodate the 4-fluorophenyl-triazole moiety, which would be consistent with the observed enthalpy change between TDG and TAZTDG (ΔΔH_TAZTDG-TDG_ = −4.5 and −2.4 kcal/mol for galectins-1 and -7, respectively). However, the enthalpy change between TD139 and TAZTDG is trivial (ΔΔH_TD139-TAZTDG_ = −0.6 and 0.7 kcal/mol for galectins-1 and -7, respectively) because the additional 4-fluorophenyl-triazole is not perfectly situated in B subsite.

### Structural basis of ligand binding in solution revealed by NMR spectroscopy

The NMR assignments of galectins-1, -3 and -7 have been reported previously^31^,^32^,^33^,^34^, which serve as the basis of high resolution NMR analysis to investigate their interactions with ligands. We first carried out NMR titration of TAZTDG with galectins-1, -3 and -7 to map the binding sites onto the X-ray crystal structures using chemical shift perturbations observed in the heteronuclear ^15^N-^1^H correlation spectra. The results reflected the binding events in close proximity to the backbone amides of most amino acid residues and the side-chain indole (the heterocyclic aromatic amine) of a key β-galactoside-recognition Trp residue (Trp68^hGal1^, Trp181^hGal3^ and Trp69^hGal7^) to which the ^15^N-^1^H correlations correspond. In the case of galectin-7, the chemical shift perturbations could be extracted by monitoring the crosspeak positions as a function of TAZTDG concentration, although coalescence is observed for several crosspeaks with intermediate ligand-to-protein ratios, leading to the unfavorable line broadening during titration (Figure S4). In the case of galectins-1 and -3, however, the low μM binding affinity of TAZTDG shifts the NMR time scale to the slow exchange regime^35^; in other words, the addition of tight-binding ligands would result in loss of signals of the apo form with concomitant increase of the bound form signals with distinct chemical shifts that cannot be traced by following the movements of crosspeak positions.

The analysis of biolayer interferometry was found to be supportive of the aforementioned binding kinetics. The result showed that very slow off rates (*k*_*off*_) were observed in the bindings of galectins-1 and -3 (Figure S5), while the on and off rates were too fast to be deduced in those of galectin-7. We therefore obtained most of the bound-form assignments by recording an independent set triple resonance (^1^H, ^13^C and ^15^N) NMR experiments in the presence of TAZTDG, followed by standard sequential assignments in comparison with the apo form spectra. TAZTDG-bound NMR spectra of galectins-1 and -3 contain many more crosspeaks than those of their apo and TD139-bound form (Figures S6 and S7), suggesting the presence of multiple conformations of galectins upon binding to TAZTDG. Indeed, structural mapping of the spatial distribution of the backbone amides exhibits significant peak doubling in galectin-3, indicating that nearly the entire β-sheet of the CRD exhibits two sets of distinct crosspeaks. Additionally, subsites A and B display more spectral difference in the peak doubling as compared to subsites D and E (Figure S8). Similar peak doubling is observed for TAZTDG-bound galectin-1, but the intrinsic broad linewidths of dimeric galectin-1 are about twice the size of galectin-3, making it more difficult to have a comprehensive structural mapping of the structural polymorphism.

Given that arginine-arene interactions play an important role in the binding of TD139-like ligands^36^, we therefore assigned most of the observed ^15^N_ε_-^1^H_ε_ correlations of the Arg side-chains of galectins-1 and -3 (Figures S6 and S7), which were not available in the previously reported assignments at physiological pH^31^,^32^,^33^,^34^. Arginine side-chain ^15^N_ε_-^1^H_ε_ correlations offer the advantage that the dynamics are less restricted by the high molecular weight of proteins. Nevertheless, severe broadening is one disadvantage since the labile protons are prone to exchange with bulk solvent^37^. Although lowering the sample temperature is able to recover some signals but it inevitably slows down the solvent exchange process, the reduced overall tumbling makes it impossible to obtain sufficient signals in the HNCACB experiments for getting the C_δ_ and C_γ_ chemical shifts within the same spin system. The assignment of key arginine side-chains is confirmed by site-directed mutagenesis. For instance, R73K-hGal1 mutant was prepared for NMR study, which results in the disappearance of the corresponding ^15^N_ε_-^1^H_ε_ correlations. While the side-chain crosspeak of Arg48^hGal1^ at the subsite C is well-defined in TAZTDG-bound form, that of Arg73^hGal1^ at subsite E is much broader, suggesting a much higher degree of structural heterogeneity. It is thus possible that the fluorophenyl-triazole of TAZTDG can either occupy subsites E or B (Figure S6). Furthermore, the indole of Trp68^hGal1^ exhibits two distinct ^15^N_ε_-^1^H_ε_ correlations in the TAZTDG-bound NMR spectrum, one of which coincides with that observed in the TD139-bound spectrum, indicating that the thio-digalactoside of TAZTDG can bind to Trp68^hGal1^ in two distinct configurations and one of the two is similar to that of the TD139-bound form (Figure S6). In the case of galectin-3, the highly resolved ^15^N-^1^H correlations enable unambiguous assignments of most doublets of the TAZTDG-bound form (Figure S7). One of the two can be nicely superimposed with those of the TD139-bound form, while the other is close to those of the apo-form. There is one exception about Trp181^hGal3^ and Arg186^hGal3^ that are in direct contact with TAZTDG at subsites C and E, respectively. Their side-chain crosspeaks display large chemical shift differences that are approximately half of the TD139-bound form (Figure S7). Notably, while the side-chain ^15^N_ε_-^1^H_ε_ correlations of Arg186^hGal3^ is very broad in the absence of ligand, it becomes well-defined upon the addition of TAZTDG or TD139. The significant change is attributed to the stablising arginine-arene interaction at subsite E that effectively reduces solvent exchange. In contrast, the side-chain ^15^N_ε_-^1^H_ε_ correlations of Arg144^hGal3^, which is involved in another arginine-arene interaction at subsite B according to the X-ray crystal structure, cannot be observed even in the presence of TD139.

**Figure 3:**
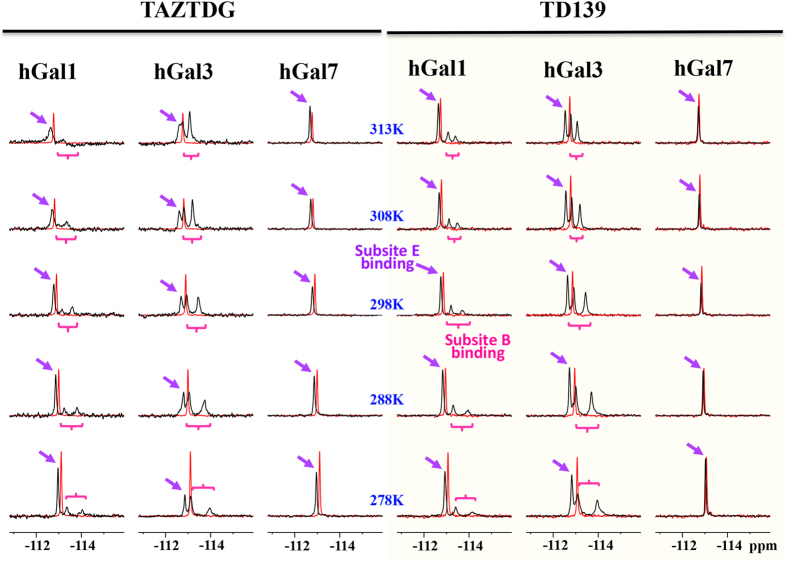
^19^F-NMR spectra of TAZTDG and TD139 in complex with galectins-1, -3 and -7. ^19^F-NMR spectra of TAZTDG (left panel) and TD139 (right panel) in complex with galectins-1, -3CRD and -7 are shown in the order of decreasing sample temperatures (from top to bottom, indicated in the middle). The ^19^F-NMR spectra of free TAZTDG and TD139 under the same conditions are shown in red as references. Distinct ^19^F resonances of the complexes were tentatively assigned, based on the complex structure analyses as detailed in the text, to the fluorine atoms binding at subsites B (purple arrows) and E (pink braces), respectively. Non-equivalency of the two broader up-field resonances was due to the binding to subsite B (see main text for explanation).

To further examine the interactions of TDG derivatives with galectins-1, -3 and -7, we exploited the high sensitivity and low background of ^19^F-NMR to monitor changes associated with the microenvironments of 4-fluorophenyl-triazole of TAZTDG and TD139 in complex with galectins. In the absence of the binding partners, both TAZTDG and TD139 display a single resonance at −113 ppm as a result of rapid tumbling in solution rendering to favorable line narrowing of the observed chemical shifts (red spectra in Fig. 3). The addition of galectin-1 and galectin-3 results in similar ^19^F-NMR spectra with three distinct resonances of broader linewidths compared to that of free TD139 (Fig. 3 right panel). In contrast, TAZTDG and TD139 exhibit a single broadened resonance when in complex with galectin-7 (Fig. 3). Linewidths of all the bound-form resonances were determined as a function of sample temperature to assess the changes in ligand dynamics according to their line broadening effects (Figure S9). These observations are consistent with the resolved X-ray structures and the ITC data of galectins-1 and -3 that both 4-fluorophenyl substituents of TD139 make extensive interactions with residues at both subsites B and E (including π-arginine, van der Waals interactions and fluorine bondings) (Fig. 2d,e), while in galectin-7 only the 4-fluorophenyl-triazole at subsite E, but not subsite B, forms a π-arginine interaction (Fig. 2f,g). It is known that one unique ^19^F resonance corresponds to one binding mode of the fluorophenyl-triazole moiety. Tentative assignments of the distinct ^19^F resonances presented in the spectra of TD139 complexes were made based on the structural knowledge regarding the different chemical environments at the binding subsites in close proximity to the fluorine atoms. First, the most down-field shifted resonance (δ < −113 ppm) in the ^19^F spectrum likely corresponds to the binding with subsite E, given that all three galectins share the common π-arginine interaction at subsite E, and all of the terminal fluorine atoms are situated at the deshielding region that on the top of the extended π-electron surface^36^, which is made up of the Arg and Asp/Glu side-chains (the right panel of Fig. 2e,g), leading to the observed down-field shifts in peak positions. Next, the two broader up-field shifted ^19^F resonances (with similar temperature-dependent linewidths broadening behavior and specific in the case of galectins-1 and -3 (Fig. 3 and Figure S9)) are assigned to two distinct binding modes to both subsite B of galectins-1 and -3. The up-field shifts are attributed to the shielding effect of the multiple orthogonal fluorine bondings on the fluorine atome at subsite B (Fig. 2d). The doublets are attributed to the dense packing by the surrounding amino acid residues of galectins-1 and -3 (Fig. 2d), reminiscent of the previously reported complicated ^19^F resonance patterns when the fluorine atom is buried in the protein interior^38^. The significantly broader linewidths also suggest the presence of slow conformational inter-conversion of the fluorinated benzyl rings of TAZTDG and TD139 on the NMR timescale. Consistent with hypothesis, the linewidths of up-field shifted ^19^F resonances of TAZTDG are much broader than those of TD139 due to the weaker binding affinties towards galectins-1 and -3.

Particularly, there are three distinct ^19^F resonances when mono-substituted TAZTDG is bound with galectins-1 and -3 (Fig. 3 left panel). The signal patterns are similar to those observed in the TD139 complexes, indicating that the fluorophenyl-triazole extension of TAZTDG can bind to both B and E subsites in solution in a similar manner as di-substituted TD139 to simultaneously occupy both sites (Figure S10). The area covered by each distinct resonance in the spectra of TAZTDG complexes also reveals that TAZTDG prefers the subsite E-binding mode when in complex with galectins-1 and -7, in agreement with the complex structures previously mentioned. Interestingly the subsite B-binding mode is dominant in the case of galectin-3. These galectin-specific binding modes are further supported by additional X-ray crystal structures of TAZTDG in complex with galectins-3 and -7 (Figure S11).

Moreover, the weak binding and hence fast averaging of free and bound signals probably explain the main reason why there is one resonance for TD139 and TAZTDG in complex with galectin-7. The resulting signal reflects an apparent population-weighted chemical shift. This also elucidates why TD139 exhibits a much smaller chemical shift difference between free and galectin-7-bound form as compared to TAZTDG because the additional substituent may not show significant chemical shift difference for the binding to subsite B.

## Conclusions

In summary, with integration of ITC, X-ray crystallography and NMR studies, we have the findings formerly undocumented that subsite E of galectins-1 and -7 is able to contribute more binding interactions than subsite B. The in-depth understanding is useful for the development of potent and selective inhibitors. For instance, because TD139 binds well with galectins-1 and -3, optimisation of C3-substitutent will possibly enhance the binding interactions at subsite B of galectin-1 to come up with more selective inhibitors for galectin-1 than for galectin-3.

## Materials and Methods

### Preparation of galectins and inhibitors

Human galectins-1, -3CRD and -7 were produced in *E. coli* according to previous studies^11^. TDG, TAZTDG and TD139 were synthesised according to the U.S. Patent Application Publication (No. 2014/0011765 A1) with several modified procedures and will be published elsewhere.

### Isothermal titration calorimetry (ITC)

Samples for use in ITC were diluted to appropriate concentrations in dialysate buffer (25 mM Tris-HCl pH 8.0, 300 mM NaCl and 5 mM β-mercaptoethanol) saved from the ultrafiltration step. All samples were filtered with 0.22 μm cutoff filters (Millipore) and extensively degassed with stirring prior to use. ITC was performed using MicroCal Auto-iTC200 (MicroCal, INc., Northampton, MA) at 298 K. TDG, TAZTDG and TD139 were dissolved in a stock solution of DMSO. To avoid heating effects due to differing concentration of DMSO in the injectant and protein solutions, 5% DMSO was added to the protein. Software provided by Microcal was used for the curve fitting of the experimental data as well as for calculation of the thermodynamic data. Specifically, all ITC data were corrected for the heat of dilution of the titrant by subtracting the excess heats at high molar ratios of ligands to galectins. Binding stoichiometry, enthalpy and equilibrium association constants were determined by fitting the corrected data to a bimolecular interaction model and summarised in Table 1.

### Crystallisation and data collection

Crystals of recombinant human galectins-1, -3CRD and -7 were grown at room temperature (298 K) using the hanging-drop vapor diffusion method from 2 μL protein solution and 2 μL reservoir solutions consisting of 0.1 M Tris pH 8.5, 0.2 M MgCl_2_, 30% (w/v) PEG 3350 (for galectin-1), 0.1 M Tris pH 8.0, 0.2 M Li_2_SO_4_, 30% (w/v) PEG 4000 (for galectin-3CRD), and 0.22 M Mg acetate, 21% (w/v) PEG 3350 (for galectin-7). The galectin-TD139 and galectin-TAZTDG complexes were obtained by soaking 20 mM TD139 and TAZTDG compounds into the preformed galectin crystals for more than a week. The reservoir solutions supplemented with 10 to 20% glycerol were used for cyroprotection of the complex crystals. Crystals were then flash-frozen in liquid nitrogen and stored for synchrotron-radiation data collection. The diffraction data were processed using the HKL2000 program suite^39^.

### Determination and refinement of the crystal structures

The crystal structures of all complexes were solved by molecular replacement with the PHENIX AutoMR^40^ using previously published ligand-free galectin structures as the starting search models: PDB entries 1W6N^41^, 2NMN^42^ and 1BKZ^43^ for galectins-1, -3CRD and -7. Model building was performed with PHENIX AutoBuild^40^. The resulting electron density maps were of good quality to show clearly the densities belonging to the bound TD139 molecules. Coordinates of TD139 and TAZTDG molecules were built into the density by using Coot^44^. Structures then underwent rounds of manual model rebuilding and refinement with Coot and PHENIX. Detailed refinement parameters are listed in supporting information Table S1. The figures were generated in Pymol^45^.

### NMR spectroscopy

To assign the observed arginine side-chain ^15^N_ε_-^1^H_ε_ correlations, uniformly ^13^C- and ^15^N-labelled galectin-1 (3.1 mM) and galectin-3CRD (1.8 mM) in phosphate buffer saline (Sigma-Aldrich, USA) were used to extract the chemical shifts of the C_δ_ and C_γ_ atoms within the same spin systems by recording high-resolution HNCACB spectra at 298 K using a Bruker AVANCE III 600 (14.1 Tesla) NMR spectrometer and a Bruker AVANCE III 850 (20.0 Tesla) NMR spectrometer, respectively. To simultaneously cover the main chain amide and arginine side-chain ^15^N chemical shifts, a very wide spectral width of 60 ppm and a carrier frequency of 106 ppm along the ^15^N dimension were used^35^. 160 complex points were collected for both indirect dimensions, i.e., ^13^C and ^15^N, to ensure sufficient spectral resolution, and a non-uniform sampling (NUS) data collection scheme was employed to collect only 10% of the expected data points, followed by Fourier transformations using default settings within Topspin 3.1 (Bruker Biospin, Germany). Previously reported NMR assignments of human galectin-1 (BMRB entry 15800) and -3 (BMRB entry 4909) were used to extract the chemical shifts of individual C_δ_ and C_γ_ atoms of arginines based on which the ^13^C_δ_-^15^N_ε_-^1^H_ε_ and ^13^C_γ_-^15^N_ε_-^1^H_ε_ correlations of the HNCACB spectra were assigned.

NMR titrations experiments were carried out using a Bruker AVANCE III 600 NMR spectrometer at 298 K with 3 mm MATCH tubes, which require only 180 μL of sample in volume. Aliquots of TAZTDG or TD139 dissolved in DMSO were added into the uniformly labelled galectins-1, -3CRD or -7 (both are 200 μM in concentration) to record a series of ^15^N-^1^H correlation spectra (fast-HSQC^46^ for galectins-1 and -7 and SOFAST-HMQC^47^ for galectin-3CRD) until the systems were saturated, i.e., no chemical shift changes were observed for the backbone amide ^15^N-^1^H correlations as shown in the Figure S4. The resulting NMR spectra were processed by NMRPipe^48^ and subsequently analysed by Sparky^49^. The resulting TAZTDG and TD139-bound galectins-1, -3CRD and -7 samples were subjected to one dimensional ^19^F-NMR spectra collection at 278, 288, 298, 308, and 313 K using a Bruker AVANCE 500 NMR spectrometer equipped with a cryogenically cooled QNP probe. Meanwhile, 1 mM of TAZTDG and TD139 were used to record reference ^19^F-NMR spectra in the absence of galectins. The linewidths of individual resonances in the ^19^F-NMR spectra were extracted by fitting the observed NMR spectra to Lorentzian line shapes using Topsin 3.1 (Bruker Biospin, Germany) and plotted as a function of temperature by Prism (GraphPad Software, Inc. USA).

## Additional Information

**Accession codes**: Structural coordinates are fully available from the Protein Data Bank using the accession codes, 4Y24, 5H9P and 5H9Q for TD139 in complex with galectins-1, -3CRD and -7, respectively; while 5H9R and 5H9S for TAZTDG in complex with galectins-3CRD and -7.

**How to cite this article**: Hsieh, T.-J. *et al*. Dual thio-digalactoside-binding modes of human galectins as the structural basis for the design of potent and selective inhibitors. *Sci. Rep.* **6**, 29457; doi: 10.1038/srep29457 (2016).

## Supplementary Material

Supplementary Information

## Figures and Tables

**Figure 1:**
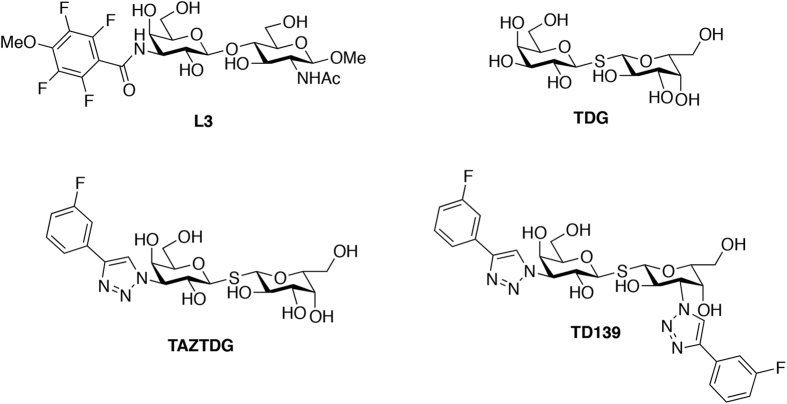
Chemical structures of L3, TDG and other derivatives.

**Table 1:** Thermodynamic binding parameters of galectins-1, -3 and -7 with TDG and its derivatives at 298 K.

Compounds	*K*_*d*_ (μM)	Relative affinity	ΔG (kcal/mol)	ΔH (kcal/mol)	-TΔS (kcal/mol)	n
	Galectin-1					
TDG	67.3 ± 8.6	1	−5.7 ± 0.08	−11.6 ± 0.26	5.9 ± 0.27	1.02 ± 0.10
TAZTDG	3.24 ± 0.3	20.7	−7.5 ± 0.05	−16.1 ± 0.51	8.6 ± 0.51	1.06 ± 0.01
TD139	0.22 ± 0.05	306	−9.1 ± 0.16	−16.7 ± 0.40	7.6 ± 0.43	0.96 ± 0.04
	Galectin-3					
TDG	75.4 ± 8.41	1	−5.6 ± 0.06	−9.4 ± 0.48	3.7 ± 0.48	1.02 ± 0.05
TAZTDG	0.84 ± 0.41	89.7	−8.3 ± 0.25	−14.7 ± 0.99	6.4 ± 1.02	0.92 ± 0.08
TD139	0.068 ± 0.01	1109	−9.9 ± 0.37	−18.2 ± 0.82	8.3 ± 0.90	0.96 ± 0.01
	Galectin-7					
TDG	572.7 ± 57.7	1	−4.4 ± 0.06	−7.6 ± 0.55	3.2 ± 0.55	0.94 ± 0.02
TAZTDG	87.0 ± 3.21	6.6	−5.5 ± 0.01	−10.0 ± 0.04	4.4 ± 0.04	0.89 ± 0.01
TD139	38.0 ± 0.71	15.1	−6.1 ± 0.06	−9.3 ± 0. 07	3.2 ± 0.09	0.99 ± 0.10
