# Supplementary material for: Dual thio-digalactoside-binding modes of human galectins as the structural basis for the design of potent and selective inhibitors
Source: Sci Rep. 2016 Jul 15;6:29457. doi: 10.1038/srep29457 (PMC4945863; doi:10.1038/srep29457)
Supplement: Supplementary Information [file srep29457-s1.pdf]

Supplementary Information

Dual thio-digalactoside-binding modes of human galectins as the structural basis for the design of potent and selective inhibitors **

Tung-Ju Hsieh, Hsien-Ya Lin, Zhijay Tu, Ting-Chien Lin, Shang-Chuen Wu, Yu-Yao Tseng, Fu-Tong Liu, Shang-Te Danny Hsu and Chun-Hung Lin**

Experimental Section

Protein preparation. Using a standard PCR-based cloning strategy, the coding regions of the full-length galectins-1 (residues 1-135), 7 (residues 1-136) and CRD domain of galectin-3 (residues 113-250) were generated and inserted into modified pET-15b or pET-28a vector (Novagen) with in-frame N- and C-terminal 6xHis-tag, respectively. All the proteins were produced in *Escherichia coli* BL21 (DE3), with 0.5 mM IPTG induction for 16 h at 20 °C. Recombinant proteins were purified by Ni^{2+}-affinity and size-exclusion chromatography to homogeneity. Purified galectins-1, -3CRD and -7 were then stored in gel-filtration buffer (25 mM Tris-HCl pH8.0, 300 mM NaCl and 5 mM β-mercaptoethanol) and concentrated to ~ 4, 20 and 9 mg/mL as determined by Bradford method for both studies of isothermal titration calorimetry and protein crystallisation.

Crystallisation and data collection. Crystals of recombinant human galectins-1, -3CRD and galectin-7 were grown at room temperature (298 K) using the hanging-drop vapor diffusion method from 2 μL protein solution and 2 μL reservoir solutions consisting of 0.1 M Tris pH 8.5, 0.2 M $MgCl_2$, 30% (w/v) PEG 3350 (for galectin-1), 0.1 M Tris pH 8.0, 0.2 M Li_2SO_4, 30% (w/v) PEG 4000 (for galectin-3CRD), and 0.22 M Mg acetate, 21% (w/v) PEG 3350 (for galectin-7). The galectin-TD139 and galectin-TAZTDG complexes were obtained by soaking 20 mM TD139 and TAZTDG into the preformed galectin crystals for more than a week. The reservoir solutions supplemented with 10 to 20% glycerol were used for cyroprotection of the complex crystals. Crystals were then flash-frozen in liquid nitrogen and stored for synchrotron-radiation data collection. The diffraction data were processed using the HKL2000 program suite[1].

Determination and refinement of the crystal structures. The crystal structures of all complexes were solved by molecular replacement with PHENIX AutoMR[2] using previously published ligand-free galectin structures as the starting search models: PDB entries 1W6N[3], 2NMN[4] and 1BKZ[5] for galectins-1, -3CRD and -7. Model building was performed with PHENIX AutoBuild[2]. The resulting electron density maps were of good quality and showed clearly the densities belonging to the bound TD139 molecules. Coordinates of TD139 and TAZTDG molecules were built into the density by using Coot[6]. Structures then underwent rounds of manual model rebuilding and refinement with Coot and PHENIX. Detailed refinement parameters are listed in supplementary Table 1. The figures were generated in Pymol[7].

Isothermal titration calorimetry (ITC). Samples for use in ITC were diluted to appropriate concentrations in dialysate buffer (25 mM Tris-HCl pH 8.0, 300 mM NaCl and 5 mM β-mercaptoethanol) saved from the ultrafiltration step. All samples were filtered with 0.22 nm cutoff filters (Millipore) and extensively degassed with stirring prior to use. Isothermal titration calorimetry was performed using MicroCal Auto-iTC200 (MicroCal, INc., Northampton, MA) at 298K. TDG, TAZTDG and TD139 were dissolved in a stock solution of DMSO. In order to avoid heating effects due to differing concentration of DMSO in the injectant and protein solutions, 5% DMSO was added to the protein. Software provided by Microcal was used for the curve fitting of the experimental data as well as for calculation of the thermodynamic data. Specifically, all ITC data were corrected for the heat of dilution of the titrant by subtracting the excess heats at high molar ratios of ligands to galectins. Binding stoichiometry, enthalpy and equilibrium association constants were determined by fitting the corrected data to a bimolecular interaction model.

NMR spectroscopy. To assign the observed arginine side-chain $^{15}N_{\varepsilon}$-$^{1}H_{\varepsilon}$ correlations, uniformly ^{13}C- and ^{15}N-labelled galectin-1 (3.1 mM) and -3CRD (1.8 mM) in phosphate buffer saline (Sigma-Aldrich, U.S.A.) were used to extract the chemical shifts of the Cδ and Cγ atoms within the same spin systems by recording high-resolution HNCACB spectra at 298K using a Bruker AVANCE III 600 (14.1 Tesla) NMR spectrometer and a Bruker AVANCE III 850 (20.0 Tesla) NMR spectrometer, respectively. To simultaneously cover the main chain amide and arginine side-chain ^{15}N chemical shifts, a very wide spectral width of 60 ppm and a carrier frequency of 106 ppm along the ^{15}N dimension were used[8]. 160 complex points were collected for both indirect dimensions, i.e., ^{13}C and ^{15}N, to ensure sufficient spectral resolution, and a non-uniform sampling (NUS) data collection scheme was employed to collect only 10% of the expected data points, followed by Fourier transformations using default settings within Topspin 3.1 (Bruker Biospin, Germany). Previously reported NMR assignments of human galectin-1 (BMRB entry 15800) and -3 (BMRB entry 4909) were used to extract the chemical shifts of individual C_{δ} and C_{γ} atoms of arginines based on which the $^{13}C_{\delta}$-$^{15}N_{\varepsilon}$-$^{1}H_{\varepsilon}$ and $^{13}C_{\gamma}$-$^{15}N_{\varepsilon}$-$^{1}H_{\varepsilon}$ correlations of the HNCACB spectra were assigned.

NMR titrations experiments were carried out using a Bruker

AVANCE III 600 NMR spectrometer at 298K with 3 mm MATCH tubes, which require only 180 μL of sample in volume. Aliquots of TAZTDG or TD139 dissolved in DMSO were added into the uniformly labelled galectin-1, -3CRD or -7 (both are 200 μM in concentration) to record a series of ^{15}N-^{1}H correlation spectra (fast-HSQC[9] for galectin-1 and -7 and SOFAST-HMQC[10] for galectin-3CRD) until the systems were saturated, i.e., no chemical shift changes were observed for the backbone amide ^{15}N-^{1}H correlations. The resulting NMR spectra were processed by NMRPipe[11] and subsequently analysed by Sparky[12]. The resulting TAZTDG and TD139-bound galectins-1, -3CRD and -7 samples were subjected to one dimensional ^{19}F NMR spectra collection at 278, 288, 298, 308, 313K using a Bruker AVANCE 500 NMR spectrometer equipped with a cryogenically cooled QNP probe. Meanwhile, 1 mM of TAZTDG and TD139 were used to record reference ^{19}F NMR spectra in the absence of galectins. The linewidths of individual resonances in the ^{19}F NMR spectra were extracted by fitting the observed NMR spectra to Lorentzian line shapes using Topsin 3.1 (Bruker Biospin, Germany) and plotted as a function of temperature by Prism (GraphPad Software, Inc. U.S.A.).

Biolayer interferometry. TD139 binding affinity of galectins-1 and -3CRD were quantitatively measured in 96-well microplates at 300K by Octet Red System (FortéBio) as reported procedure[13]. Specifically, biotinylated galectins were prepared according to the standard protocol (provided by FortéBio), and adjusted to final concentration of 1μM in assay buffer condition (50 mM Tris-HCl pH 7.5, 300 mM NaCl and 5 mM β-mercaptoethanol). Biotinylated galectins were then immobilised on Super Streptavidin Biosensors (FortéBio, Inc.), while free streptavidin sites on biosensor were then blocked by incubation with biocytin (10 mg/ml) to avoid non-specific interactions. The assay was carried out by placing galectin-coated biosensors into the wells with a concentration series of 3-fold diluted TD139 solutions (200 μl per well, from a top concentration of 330 nM for galectin-1 and 165 nM for galectin-3CRD) and measuring changes in layer thickness (in nanometers) of biosensors with time. Measurements were followed by 120 seconds baseline step, 60 seconds association step and 200 seconds dissociation step. Baseline and dissociation steps were carried out in assay buffer only. All the data were processed and calculated using Fortébio software. Global Analysis mode was used to derive a set of parameters including k_{on}, k_{off} and K_d values from a set of association and dissociation curves (Figure S3). Two additional parallel Super Streptavidin biosensors were coated with

biotinylated galectin and biocytin, separately, and only incubated with assay buffer as double reference controls.

Protein structure accession numbers. The atomic coordinates and structure factors have been deposited in the PDB under accession codes, 4Y24, 5H9P and 5H9Q for TD139 in complex with galectins-1, -3CRD and -7, respectively, and 5H9R, 5H9S for TAZTDG in complex with galectins-3CRD and -7.

References:

1 Otwinowski, Z. & Minor, W. in *Methods in Enzymology* Vol. Volume 276 (ed Charles W. Carter, Jr.) 307-326 (Academic Press, 1997).

2 Adams, P. D. *et al.* PHENIX: a comprehensive Python-based system for macromolecular structure solution. *Acta Cryst. D***66**, 213-221 (2010).

3 Lopez-Lucendo, M. F. *et al.* Growth-regulatory human galectin-1: crystallographic characterisation of the structural changes induced by single-site mutations and their impact on the thermodynamics of ligand binding. *J Mol Biol.* **343**, 957-970 (2004).

4 Collins, P. M., Hidari, K. I. & Blanchard, H. Slow diffusion of lactose out of galectin-3 crystals monitored by X-ray crystallography: possible implications for ligand-exchange protocols. *Acta Cryst. D***63**, 415-419 (2007).

5 Leonidas, D. D. *et al.* Structural basis for the recognition of carbohydrates by human galectin-7. *Biochemistry* **37**, 13930-13940 (1998).

6 Emsley, P., Lohkamp, B., Scott, W. G. & Cowtan, K. Features and development of Coot. *Acta Cryst. D***66**, 486-501 (2010).

7 The PyMOL Molecular Graphics System, Version 1.8. Schrödinger, LLC.

8 Cavangh, J., Skelton, N. J., Fairbrother, W. J., Rance, M. & Palmer III, A. G. *Protein NMR spectroscopy: principles and practice*. 2 edn, (Elsevier, 2006).

9 Hwang, T. L., van Zijl, P. C. & Mori, S. Accurate quantitation of water-amide proton exchange rates using the phase-modulated CLEAN chemical EXchange (CLEANEX-PM) approach with a Fast-HSQC (FHSQC) detection scheme. *J Biomol NMR.* **11**, 221-226 (1998).

10 Schanda, P. & Brutscher, B. Very fast two-dimensional NMR spectroscopy for real-time investigation of dynamic events in proteins on the time scale of seconds. *J Am Chem Soc.* **127**, 8014-8015 (2005).

11 Delaglio, F. *et al.* NMRPipe: a multidimensional spectral processing system based on UNIX pipes. *J Biomol NMR.* **6**, 277-293 (1995).

12 Sparky v. 3.115 (University of California, San Francisco, 2008).

13 Hsieh, T. J. *et al.* Structural Basis Underlying the Binding Preference of Human Galectins-1, -3 and -7 for Galbeta1-3/4GlcNAc. *PLoS One.* **10**, e0125946, (2015).

14 Meynier, C. *et al.* NMR and MD investigations of human galectin-1/oligosaccharide

complexes. *Biophys J.* **97**, 3168-3177 (2009).

Supplementary Table 1. Data collection and refinement statistics

	**hGal1/ TD139**	**hGal3-CRD/ TD139**	**hGal3-CRD/ TAZTDG**	**hGal7/ TD139**	**hGal7/ TAZTDG**
**Data collection**					
Space grouop	$P\ 2_12_12_1$	$P\ 2_12_12_1$	$P\ 2_12_12_1$	$P\ 2_12_12_1$	$P\ 2_12_12_1$
Cell dimensions					
a, b, c (Å)	43.6,58.2,111.0	37.2, 57.1, 63.7	36.8, 57.7, 63.0	53.9, 64.4, 70.6	53.9, 64.5, 73.0
α, β, γ (°)	90, 90, 90	90, 90, 90	90, 90, 90	90, 90, 90	90, 90, 90
Resolution (Å)	30-2.32	30-2.04	30-1.58	30-1.93	30-1.82
	(2.40-2.32)	(2.11-2.04)	(1.64-1.58)	(2.00-1.93)	(1.89-1.82)
I/s	22.94 (5.5)	30.54 (4.9)	22.8 (3.32)	44.94 (11.86)	44.96 (3.95)
Completeness (%)	96.5 (86.3)	99.0 (95.1)	99.7 (98.2)	99.6 (96.5)	99.8 (100)
Redundancy	7.1 (5.3)	10.5 (7.8)	4.6 (4.4)	6.0 (6.0)	6.9 (6.9)
$R_{sym}{}^{b}$	0.092 (0.274)	0.071 (0.355)	0.060 (0.447)	0.036 (0.152)	0.033 (0.493)
**Refinement**					
$R_{work}{}^{c}$ / $R_{free}{}^{d}$	0.2004 /	0.1653 /	0.1695 /	0.1890 /	0.1829 /
	0.2479	0.1950	0.1970	0.2385	0.2274
B-factors (mean)	32.30	32.00	17.91	30.00	47.63
R.m.s. deviations					
Bond lengths (Å)	0.008	0.005	0.006	0.005	0.008
Bond angles (°)	0.97	1.025	1.22	1.001	1.142
Ramachandran (%)					
Favored (%)	97.0	99.3	98.6	97.3	97.3
Outliers (%)	0	0	0	0	0

[a]Statistics for data from the highest-resolution shell are shown in parentheses.

[b]$R_{sym} = (\Sigma\Sigma|I_{hkl} - < I >|) / (\Sigma I_{hkl})$, where the average intensity $<I>$ is taken overall symmetry equivalent measurements and I_{hkl} is the measured intensity for any given reflection.

[c]$R_{work} = (\Sigma\big||F_o| - k|F_c|\big|) / (\Sigma|F_o|)$, where F_o and F_c are the observed and calculated structure factor amplitudes, respectively.

[d]R_{free} was calculated for R factor using only an unrefined subset of reflections data (5%).

Legends of Supplementary Figures

Figure S1. **Surface representation of the CRD of human galectin-1.** The CRD consists of five subsites[14], including subsites A (shown in color of light blue), B (pale green), C (light pink), D (light orange), and E (pale cyan). Type 2 N-acetyl-lactosamine (LacNAc) is shown to illustrate its location at subsites C and D.

Figure S2. **Conservation of the Arg residues located at subsites B and E in human galectins-1 to -12.** Structure-based sequence alignment of S3-S6 β-strands in human galectins-1 to -12. Secondary structures are designated according to the resolved x-ray structures. The highly conserved N-acetyl-lactosamine interacting residues among human galectins were indicated by asterisks. The two possible arene-interacting arginine residues located in subsites B and E are colored in yellow and red, respectively.

Figure S3. **Overall structures of human galectins-1, -3CRD and -7 in complex with TD139.** (a-c) Ribbon representations of TD139 (shown in yellow stick model) bound to hGal1 (a), hGal3-CRD (b) and hGal7 (c). Only one monomer exists in the asymmetric units of galectin-3CRD crystal, while asymmetric unit of galectins-1 and 7 harbor side-by-side and back-to-back symmetric dimer, respectively. All the observed CRDs

of galectins adopt a typical β-sandwich fold composed by two antiparallel β-sheets. Numbering of the β-strands of S-sheet (S1-S6) and F-sheet (F1-F5) is shown as indicated. (d-f) F_o-F_c omit electron density map (contoured at 2.5σ) of the symmetrical TD139 molecule bound to hGal1, hGal3-CRD and hGal7, respectively. To make it clear, the first galactose, which interacts with the conserved Trp residue, is indicated as GalA while the other is named GalB. Carbons 1, 3 and 5 of the Gal moieties are also labelled. Of note, two TD139 molecules were found in the galectin-1 dimer with significant distinct orientations of the fluorophenyl-triazolyl substituent at subsite E. Given the TD139 bound in chain A protomer of galectin-1 provides an additional anion-π interaction with a aspartic residue (Asp54) of subsite E and only chain B protomer of the galectin-7 contains TD139, we refer to chain A of galectin-1 and chain B of galectin-7, respectively, in the all structural analyses. (g-i) Consecutive TD139-binding subsites of galectins-1, 3 and 7 are labelled. Residues involved in TD139 recognition are highlighted with $2F_o$-F_c electron density map (contoured at 1σ). Polar interactions among galectin residues and within galectin/TD139 complex are shown as gray and yellow dashed lines, respectively.

Figure S4. **Representative NMR ^{15}N-^{1}H HSQC chemical shift titration data for TAZTDG and TD139 binding to galectins-1, -3 and**

-7. Overlay of the representative $^{15}N\varepsilon$-$^{1}H\varepsilon$ correlations form the indole side-chain of Trp residues in the presence of different molar ratios of TAZTDG and TD139 over galectins-1 (the top panel of a and b), -3CRD (top panel of c and d) and -7 (top panel of e and f) as color-coded according to each concentration labelling at the bottom columns. The arrows indicate the shift of peaks during the titration process. In the case of galectin-7 in complex with TAZTDG (bottom columns of e), the chemical shift perturbations could be extracted by monitoring the crosspeak positions as a function of TAZTDG concentration featuring a fast exchange process. In the case of galectins-1 and -3CRD, however the low μM binding affinity of TAZTDG and TD139 shifts the NMR time scale to the slow exchange regime as it is revealed in the bottom columns of (a), (b), (c) and (d).

Figure S5. **Biolayer interferometry-based kinetic analysis for the binding of TD139 with galectins-1 and -3.** K_d values and kinetic parameters (k_{on} and k_{off}) of the TD139 in complex with galectins-1 and -3CRD were listed by fitting the curves of biolayer interferometry experiments at 300K. Experimental procedures were detailed as those described in Supplementary Experimental Section.

Figure S6. **^{15}N-^{1}H correlation analysis of TAZTDG and TD139**

binding to galectin-1. Overlay of the ^{15}N-^{1}H HSQC spectra of apo, TAZTDG-bound and TD139-bound galectin-1 that are shown in red, green and blue. The spectra are recorded at 14.1 Tesla, 298K with a five-fold ligand-to-protein molar ratio. The Arg side-chains in direct contact with TAZTDG and TD139, i.e., Arg48^{hGal1} and Arg73^{hGal1}, display significant chemical shift perturbations while the others are not significantly perturbed as shown in the expanded view (top right). Additionally, several crosspeaks exhibit doublets in the presence of TAZTDG (5 equiv) as highlighted for the side-chain indole of Trp68 (lower left) and a selected backbone amide crosspeak of Val59 in the lower right corner. Right panel: An expanded view of the crystal structure of galectin-1 in complex with TD139 shown in stick representation of which carbon, nitrogen, oxygen, sulfur and fluorine atoms are shown in white, blue, red, yellow and cyan, respectively. The structure of galectin-1 is shown with semi-transparent electrostatic surface representation with positively and negatively charged surfaces colored in blue and red, respectively. The identities of the ligand-contacting residues are indicated accordingly.

Figure S7. **^{15}N-^{1}H correlation analysis of TAZTDG and TD139 binding to galectin-3.** Overlay of the ^{15}N-^{1}H HSQC spectra of apo, TAZTDG-bound and TD139-bound galectin-3CRD that are shown in red,

green and blue. The spectra are recorded at 14.1 Tesla, 298K with a two-fold ligand-to-protein molar ratio. The Arg side-chains in direct contact with TAZTDG and TD139 (i.e., Arg162 and Arg186) display significant chemical shift perturbations while the others are not significantly perturbed as shown in the expanded view (top right). Similar to galectin-1, many crosspeaks, including those of Arg side-chains, exhibit doublets in the presence of TAZTDG (2 equiv) as highlighted for the side-chain indole of Trp181 (lower left) and backbone amide crosspeak of Val65 (lower right corner). Right panel: An expanded view of the crystal structure of galectin-3CRD in complex with TD139 shown in stick representation of which carbon, nitrogen, oxygen, sulfur and fluorine atoms are shown in white, blue, red, yellow and cyan, respectively. The structure of galectin-3CRD is shown with semi-transparent electrostatic surface representation with positively and negatively charged surfaces colored in blue and red, respectively. The identities of the ligand-contacting residues are indicated accordingly.

Figure S8. **Structural mapping of multiple conformations of galectin-3 upon binding to TAZTDG.** Residues that exhibit doublets in the TAZTDG-bound ^{15}N-^{1}H HSQC spectra are shown in spheres regarding their backbone amide nitrogen atoms with the identities indicated. TD139 is shown is stick representation to indicate the binding

site. The color code reflects the extent of the doublet separations. The more significant they are, the more red-shifted as shown along the rainbow spectrum.

Figure S9. **Linewidth analysis of the observed ^{19}F resonances of TAZTDG and TD139 in complex with galectins as a function of temperature.** The linewidths of TAZTDG and TD139 in the absence of galectins under the different temperatures are shown in blue as a control. When TAZTDG and TD139 in complex with galectin-1 (a and b) and galectin-3CRD (c and d), three ^{19}F resonances were measured and their corresponding linewidths are shown in black, red and green according to their chemical shifts from the most down field-shifted to the most up filed-shifted ones. For galectin-7 (e and f), only one resonance was observed and the corresponding linewidths are shown in black.

Figure S10. **Dual binding modes of human galectin inhibitors.** Human galectins represent a family of β-galactoside-binding lectins containing five subsites in the CRD. So far most structural studies focus on subsites C and D. On the basis of isothermal titration calorimetry, X-ray crystallography and NMR spectroscopy, our results indicated that subsites B and E can enhance the affinity to a different level, and that subsite E of galectins-1 and -7 contributes more binding interactions than

subsite B, which is reversed in galectin-3. One of the inhibitors, TAZTDG, was found to display dual binding modes for subsite B or E.

Figure S11. **Structures of human galectin-3CRD and galectin-7 in complex with TAZTDG.** Stereo view of hGal3-CRD (a) and hGal7 (b) in complex with the C2-asymmetric TAZTDG. The F_o-F_c omit electron density map (contoured at 2.5σ, grey) were shown for bound TAZTDG molecules, while 2F_o-F_c electron density (contoured at 1σ, blue and green, respectively) were shown for the amino acid residues of galectin-3 and 7 which involved in TAZTDG recognition. It is clear that the single 4-fluorophenyl substituent of TAZTDG favors binding to subsite B in galectin-3, while subsite E is more preferred in the case of galectin-7.

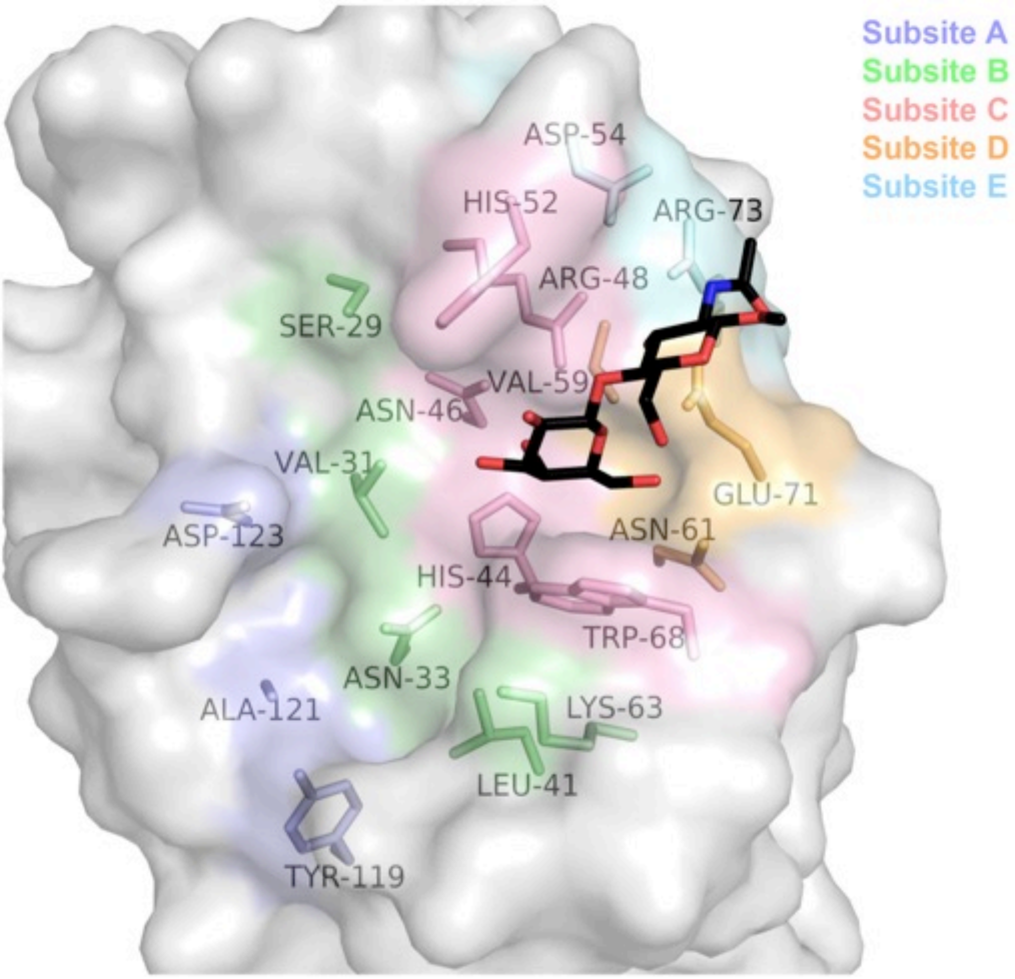
Subsite A
Subsite B
Subsite C
Subsite D
Subsite E
ASP-54
HIS-52
ARG-73
ARG-48
SER-29
VAL-59
ASN-46
VAL-31
GLU-71
ASP-123
ASN-61
HIS-44
TRP-68
ASN-33
ALA-121
LYS-63
LEU-41
TYR-119

Figure S1

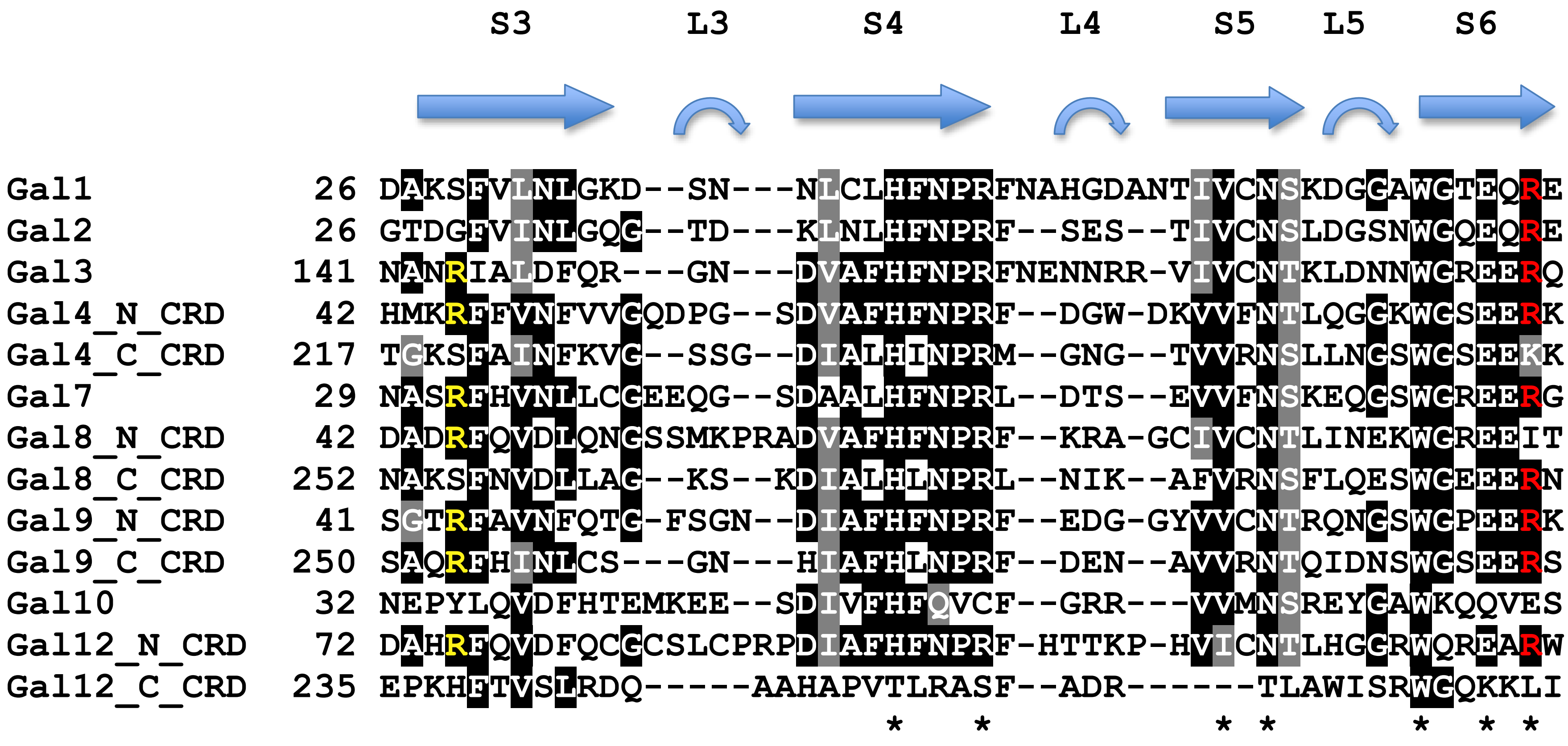
S3 L3 S4 L4 S5 L5 S6
Gal1 26 DAKSFVLNLGKD--SN---NLCLHFNPRFNAHGDANTIVCNSKDGGAWGTEQRE
Gal2 26 GTDGFVINLGQG--TD---KLNLHFNPRF--SES--TIVCNSLDGSNWGQEQRE
Gal3 141 NANRIALDFQR---GN---DVAFHFNPRFNENNRR-VIVCNTKLDNNWGREERQ
Gal4_N_CRD 42 HMKRFFVNFVVGQDPG--SDVAFHFNPRF--DGW-DKVVFNTLQGGKWGSEERK
Gal4_C_CRD 217 TGKSFAINFKVG--SSG--DIALHINPRM--GNG--TVVRNSLLNGSWGSEEKK
Gal7 29 NASRFHVNLLCGEEQG--SDAALHFNPRL--DTS--EVVFNSKEQGSWGREERG
Gal8_N_CRD 42 DADRFQVDLQNGSSMKPRADVAFHFNPRF--KRA-GCIVCNTLINEKWGREEIT
Gal8_C_CRD 252 NAKSFNVDLLAG--KS--KDIALHLNPRL--NIK--AFVRNSFLQESWGEEERN
Gal9_N_CRD 41 SGTRFAVNFQTG-FSGN--DIAFHFNPRF--EDG-GYVVCNTRQNGSWGPEERK
Gal9_C_CRD 250 SAQRFHINLCS---GN---HIAFHLNPRF--DEN--AVVRNTQIDNSWGSEERS
Gal10 32 NEPYLQVDFHTEMKEE--SDIVFHFQVCF--GRR---VVMNSREYGAWKQQVES
Gal12_N_CRD 72 DAHRFQVDFQCGCSLCPRPDIAFHFNPRF-HTTKP-HVICNTLHGGRWQREARW
Gal12_C_CRD 235 EPKHFTVSLRDQ-----AAHAPVTLRASF--ADR------TLAWISRWGQKKLI
* * * * * * *

Figure S2.

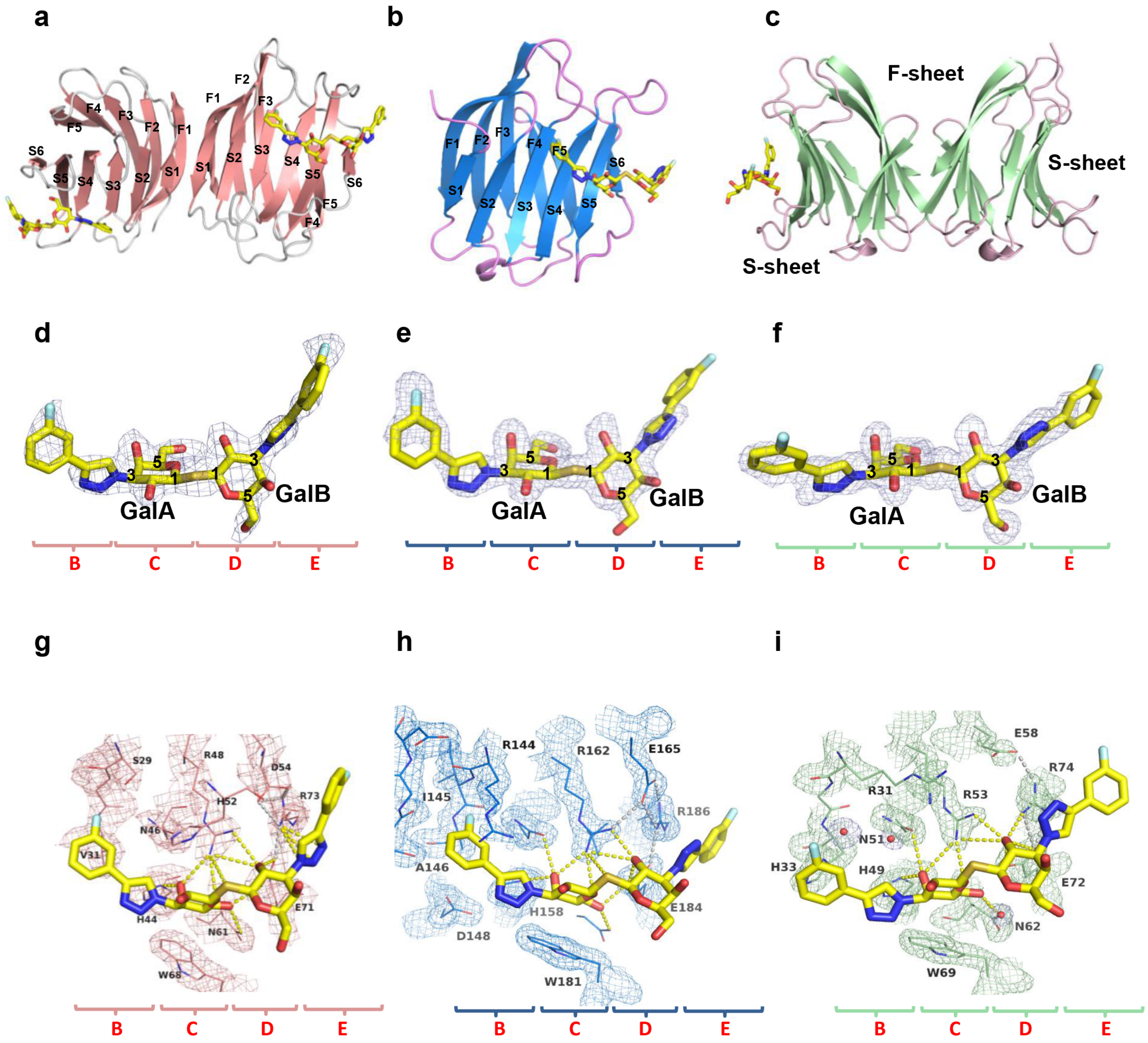
a
b
c
F-sheet
S-sheet
S-sheet
d
e
f
GalA
GalB
B
C
D
E
g
h
i
R144
R162
E165
I145
R186
A146
H158
D148
W181
E58
R74
R31
R53
N51
H33
H49
E72
N62
W69

Figure S3.

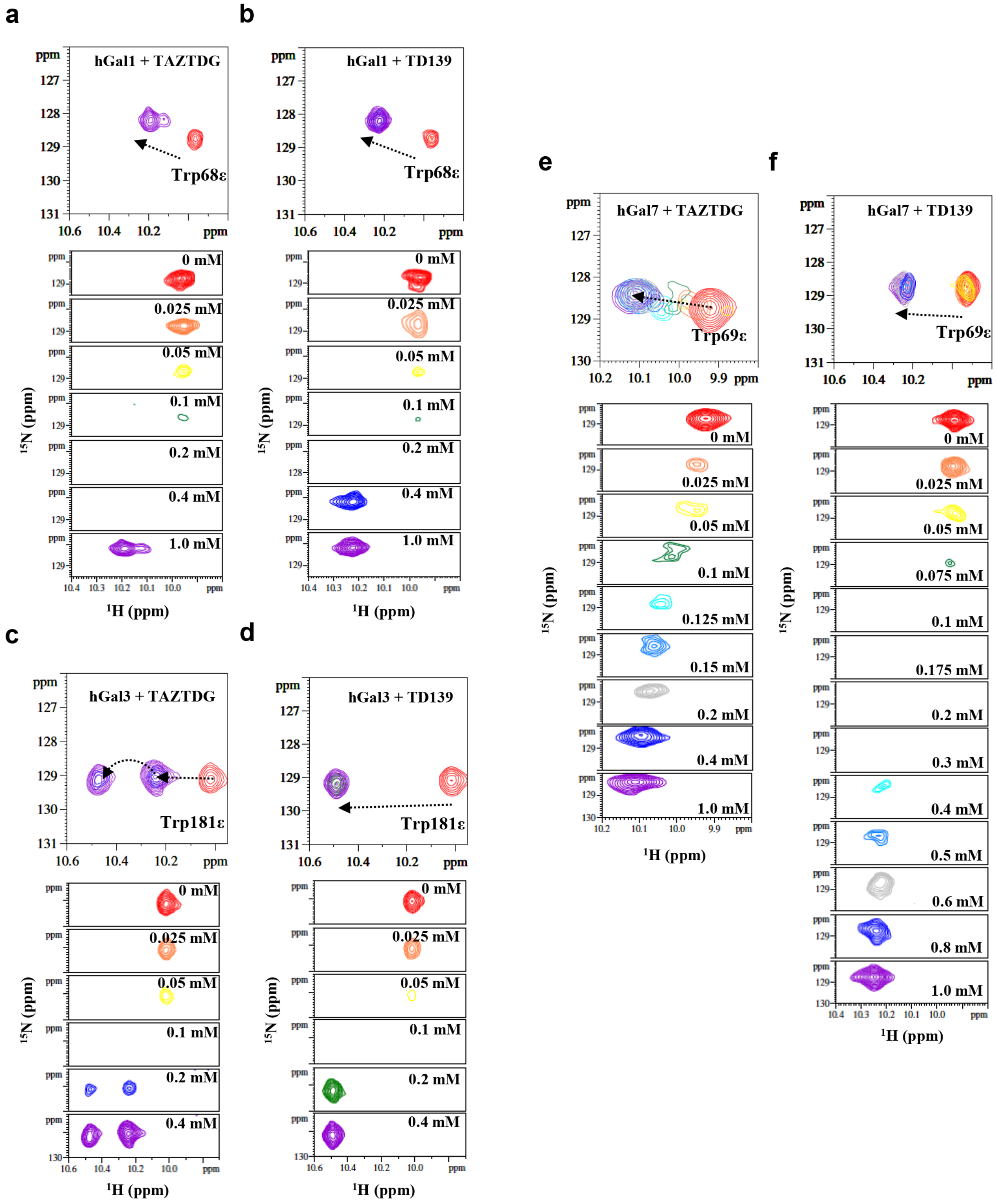
a
hGal1 + TAZTDG
Trp68ε
0 mM
0.025 mM
0.05 mM
0.1 mM
0.2 mM
0.4 mM
1.0 mM
¹⁵N (ppm)
¹H (ppm)
b
hGal1 + TD139
Trp68ε
0 mM
0.025 mM
0.05 mM
0.1 mM
0.2 mM
0.4 mM
1.0 mM
¹⁵N (ppm)
¹H (ppm)
c
hGal3 + TAZTDG
Trp181ε
0 mM
0.025 mM
0.05 mM
0.1 mM
0.2 mM
0.4 mM
¹⁵N (ppm)
¹H (ppm)
d
hGal3 + TD139
Trp181ε
0 mM
0.025 mM
0.05 mM
0.1 mM
0.2 mM
0.4 mM
¹⁵N (ppm)
¹H (ppm)
e
hGal7 + TAZTDG
Trp69ε
0 mM
0.025 mM
0.05 mM
0.1 mM
0.125 mM
0.15 mM
0.2 mM
0.4 mM
1.0 mM
¹⁵N (ppm)
¹H (ppm)
f
hGal7 + TD139
Trp69ε
0 mM
0.025 mM
0.05 mM
0.075 mM
0.1 mM
0.175 mM
0.2 mM
0.3 mM
0.4 mM
0.5 mM
0.6 mM
0.8 mM
1.0 mM
¹⁵N (ppm)
¹H (ppm)

Figure S4.

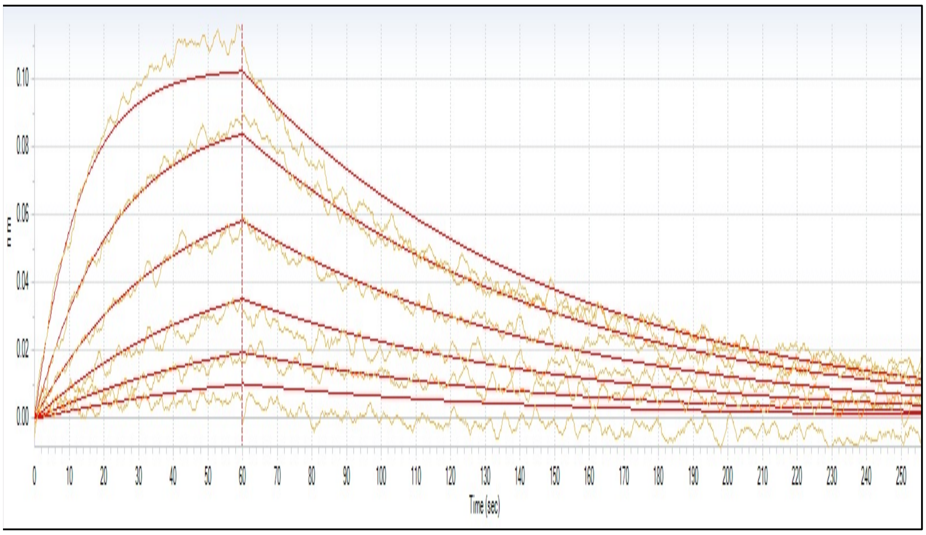

Gal-1• TD139

K_D = 56 nM
k_{on} = 1.98 X 10^5 M^{-1} s^{-1}
k_{off} = 1.1 X 10^{-2} s^{-1}
R^2 = 1.0

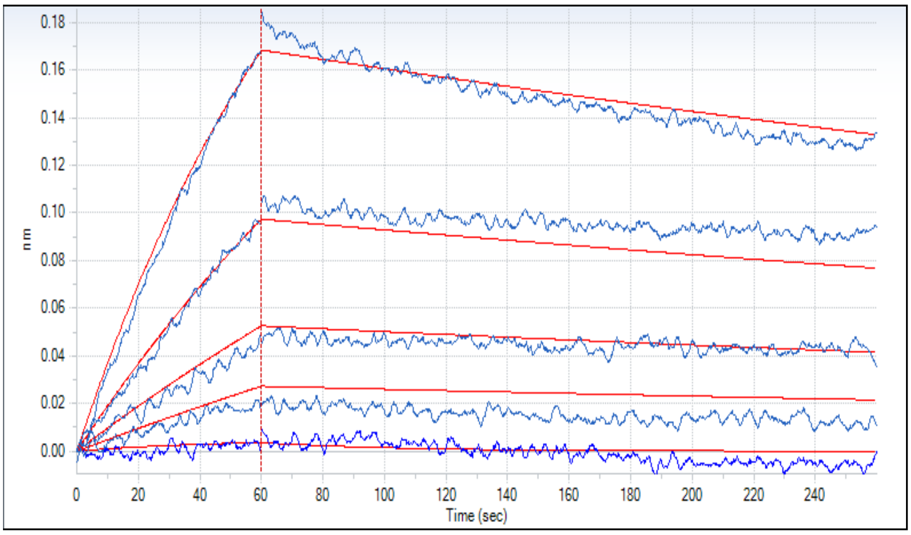

Gal-3• TD139

K_D = 18.3 nM
k_{on} = 6.49 X 10^4 M^{-1} s^{-1}
k_{off} = 1.19 X 10^{-3} s^{-1}
R^2 = 0.98

Figure S5.

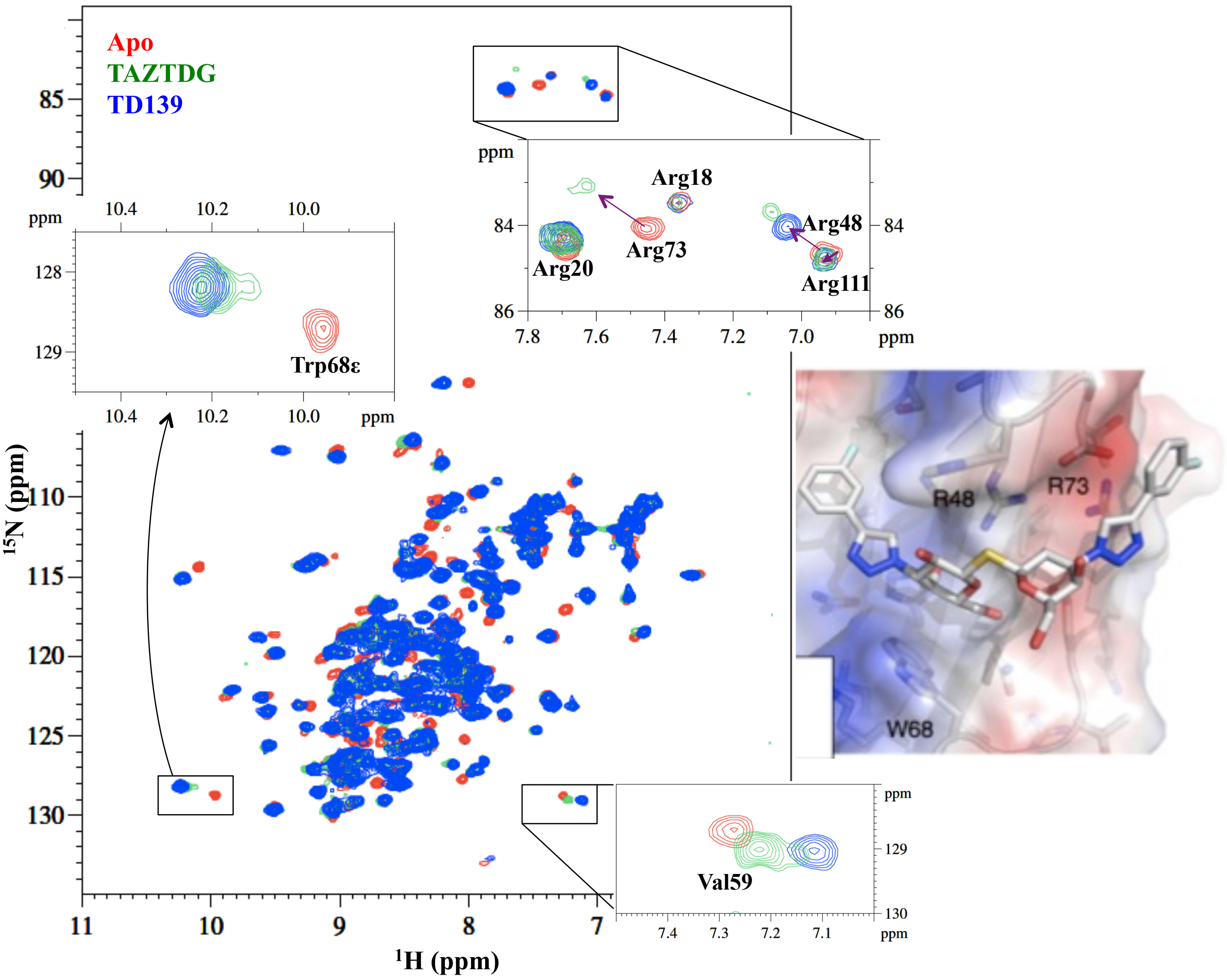
Apo
TAZTDG
TD139
ppm
Arg18
Arg73
Arg20
Arg48
Arg111
Trp68ε
R48
R73
W68
Val59
^{15}N (ppm)
^{1}H (ppm)

Figure S6.

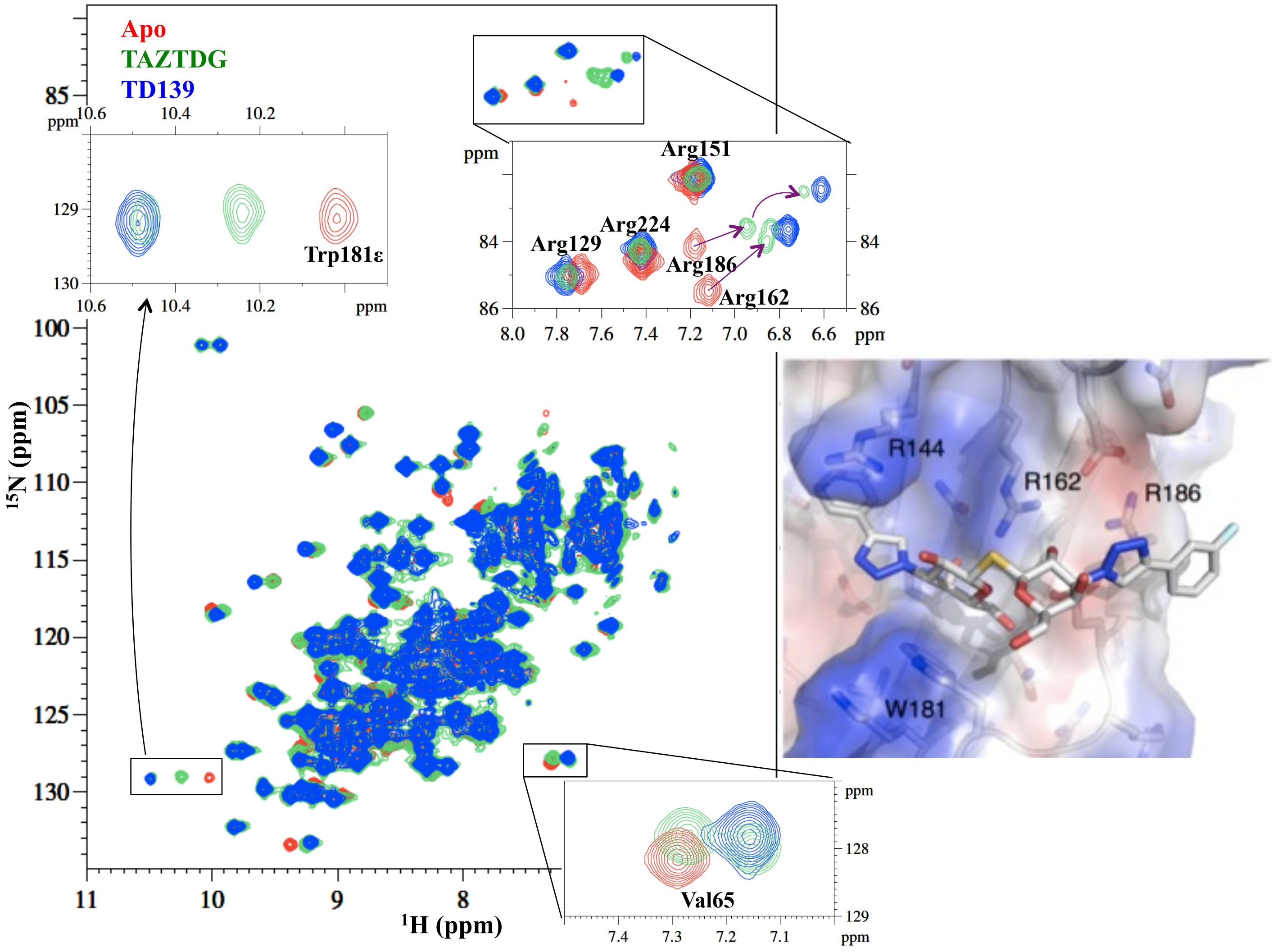

Apo
TAZTDG
TD139
Trp181ε
Arg151
Arg224
Arg129
Arg186
Arg162
Val65
R144
R162
R186
W181
15N (ppm)
1H (ppm)

Figure S7.

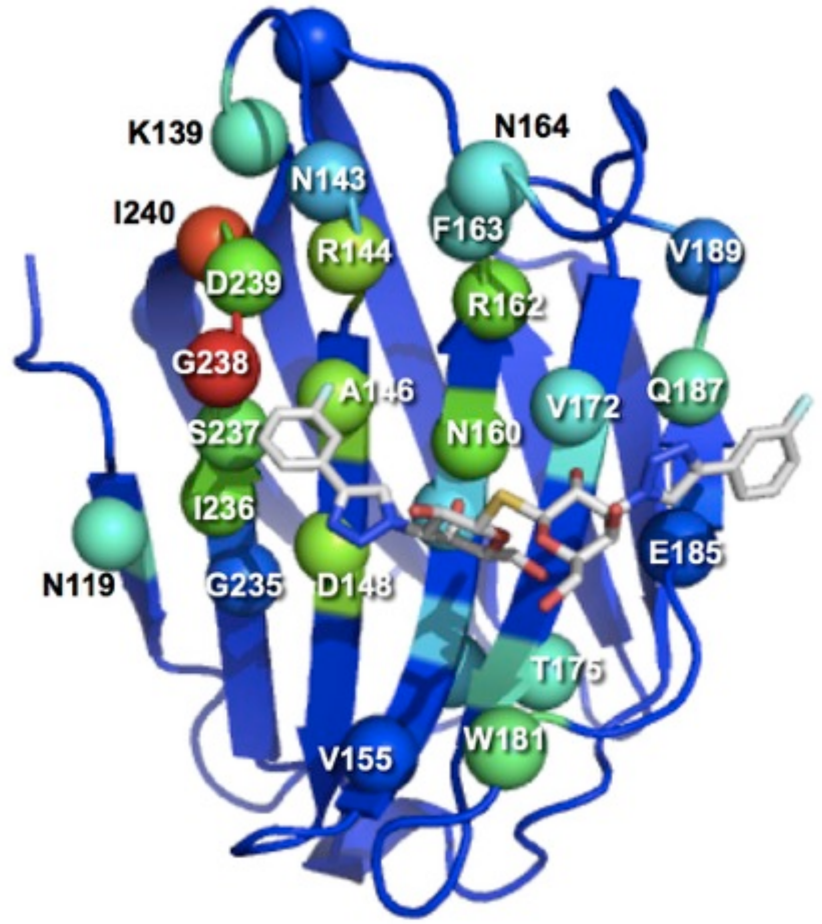
K139
N164
N143
I240
F163
R144
V189
D239
R162
G238
A146
Q187
V172
S237
N160
I236
E185
N119
G235
D148
T175
W181
V155

Figure S8.

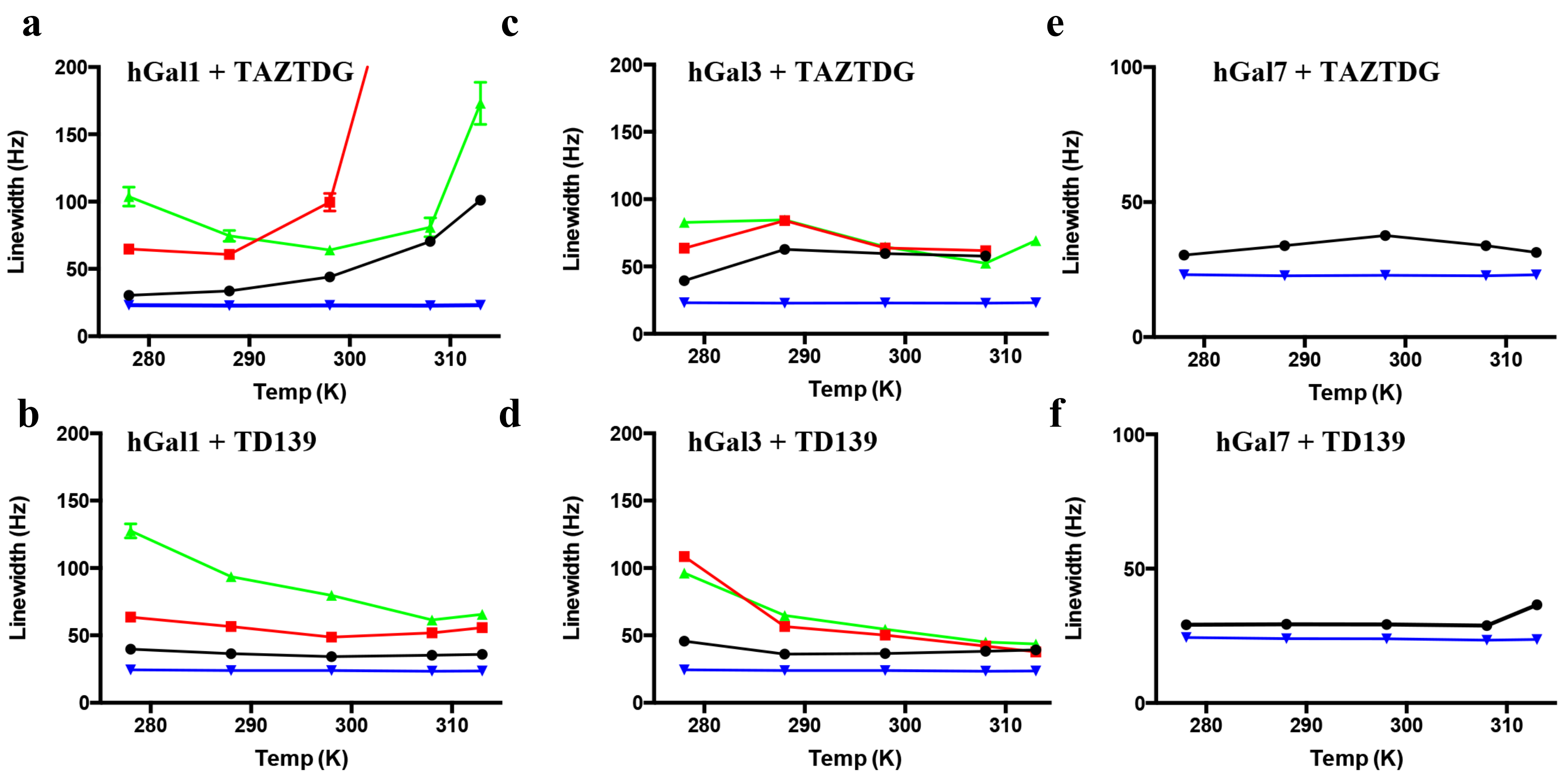
a
hGal1 + TAZTDG
Linewidth (Hz)
Temp (K)
c
hGal3 + TAZTDG
Linewidth (Hz)
Temp (K)
e
hGal7 + TAZTDG
Linewidth (Hz)
Temp (K)
b
hGal1 + TD139
Linewidth (Hz)
Temp (K)
d
hGal3 + TD139
Linewidth (Hz)
Temp (K)
f
hGal7 + TD139
Linewidth (Hz)
Temp (K)

Figure S9.

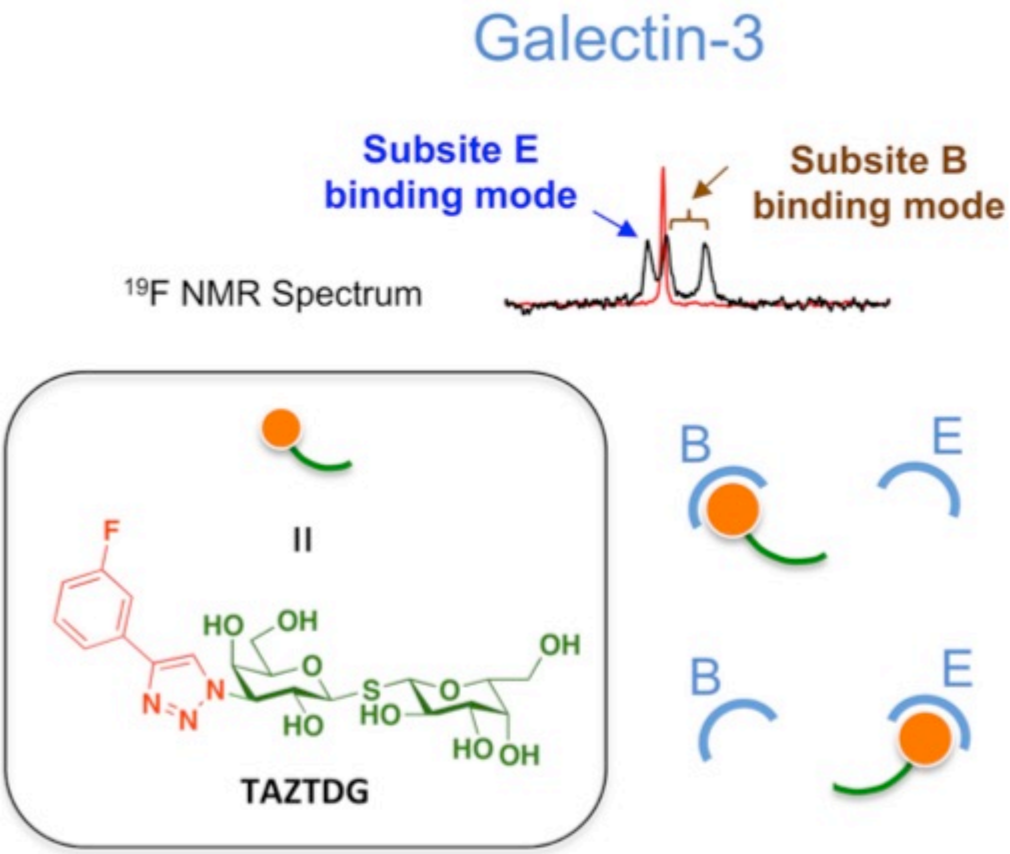

Galectin-3
Subsite E
binding mode
Subsite B
binding mode
19F NMR Spectrum
=
TAZTDG
B
E
B
E

Figure S10.

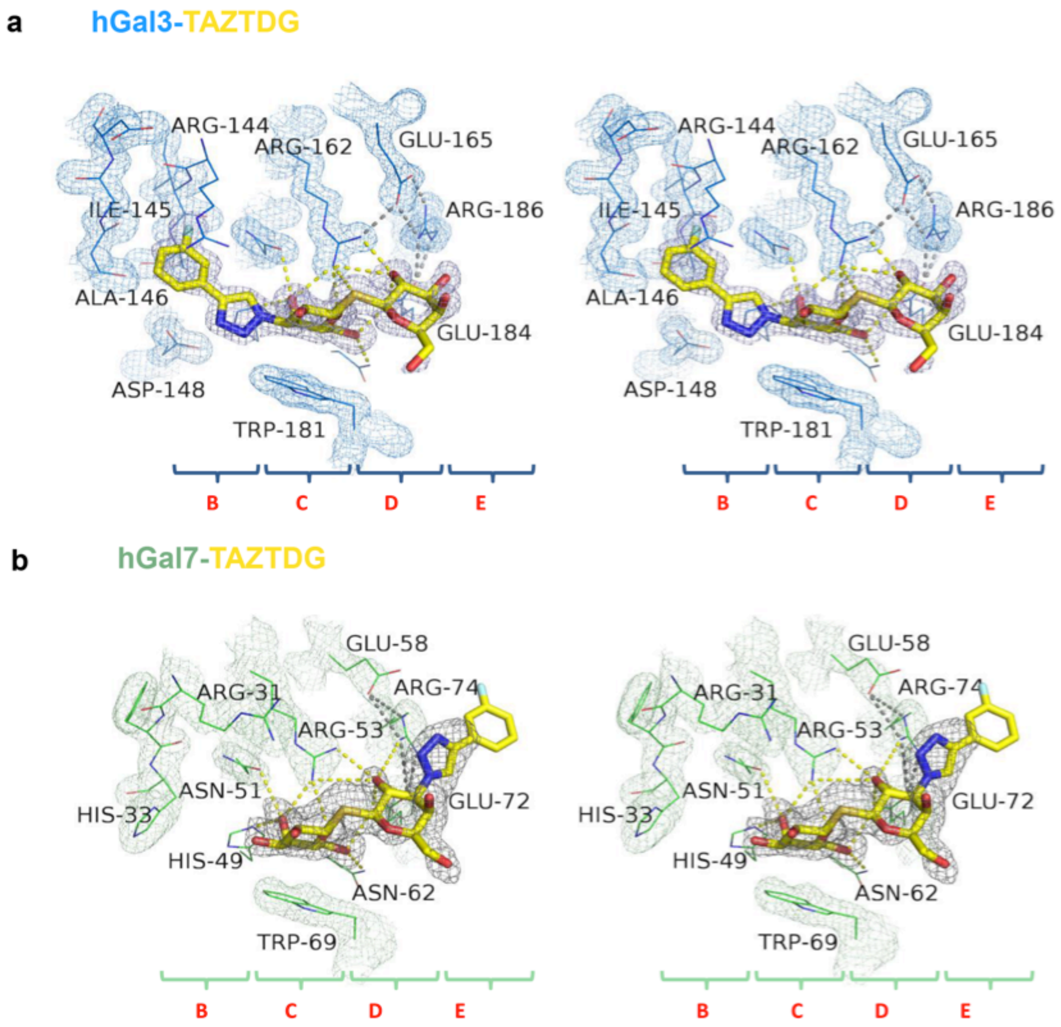

a
hGal3-TAZTDG
ARG-144
ARG-162
GLU-165
ILE-145
ARG-186
ALA-146
GLU-184
ASP-148
TRP-181
B
C
D
E
ARG-144
ARG-162
GLU-165
ILE-145
ARG-186
ALA-146
GLU-184
ASP-148
TRP-181
B
C
D
E
b
hGal7-TAZTDG
GLU-58
ARG-31
ARG-74
ARG-53
ASN-51
GLU-72
HIS-33
HIS-49
ASN-62
TRP-69
B
C
D
E
GLU-58
ARG-31
ARG-74
ARG-53
ASN-51
GLU-72
HIS-33
HIS-49
ASN-62
TRP-69
B
C
D
E

Figure S11.
